# Addressing data scarcity in CT imaging: novel approaches for the detection of radiation-induced pneumonitis

**DOI:** 10.1093/bjrai/ubaf014

**Published:** 2025-10-16

**Authors:** Sotiris Raptis, Christos Ilioudis, Kiki Theodorou

**Affiliations:** Medical Physics Department, Medical School, University of Thessaly, Larisa 41500, Greece; Department of Information and Electronic Engineering, International Hellenic University (IHU), Thessaloniki 57001, Greece; Medical Physics Department, Medical School, University of Thessaly, Larisa 41500, Greece; Biomedical Physics Department, King Faisal Specialist Hospital and Research Center, Riyadh 11211, Saudi Arabia

**Keywords:** radiation pneumonitis, lung cancer, GAN-generated images, radiomics integration, spatial attention mechanisms

## Abstract

**Objectives:**

Radiation-induced pneumonitis (RP) is a critical complication of radiotherapy in lung cancer patients, and its early detection remains a challenge due to the limited availability of annotated CT imaging data and the subtle nature of disease evolution. The objective of this study is to enhance the detection and localization of RP in CT by integrating advanced data augmentation, self-supervised learning, and synthetic data generation techniques.

**Methods:**

A conditional Generative Adversarial Network (cGAN) was used to create synthetic RP images conditioned on lung segmentation masks to create anatomically plausible data for augmenting the training sets. The pipeline was created to possess double-stage self-supervised training with hierarchical pretext tasks to achieve robust features.

**Results:**

The performance of the proposed framework, for a 5-fold cross-validation, has an average accuracy of 94.04%, precision of 92.06%, with a recall of 95.1%, an F1-score of 93.56%, and area under the curve of 95.94%.

**Conclusions:**

The model was demonstrated to possess superior performance and stability in RP detection and localization, which suggests potential clinical translation.

**Advances in knowledge:**

The paper offers a novel fusion of cGAN-generated synthetic data, spatial attention, and contrastive learning to address RP detection in limited data. Interpretability is achieved by introducing Bayesian uncertainty estimation to provide translational value in clinical practice.

## Introduction

Radiation-induced pneumonitis (RP) is a serious complication of thoracic radiotherapy; it is more common in lung cancer patients.^1^ Its incidence ranges from 5% to 15% in non-small-cell lung cancer (NSCLC), up to 10% to 30% in broader cohorts incorporating systemic therapies or modern RT techniques, and has been reported at approximately 9.4% in stereotactic body RT series.^2–4^ The subtle and heterogeneous nature of RP manifestations in CT imaging, coupled with the lack of annotated datasets, poses significant challenges for early diagnosis. Although classic supervised methods have been successful, they heavily depend on large, labelled datasets, which are challenging to obtain for RP studies due to privacy constraints, labour-intensive annotation, and the limited availability of CT scans related to RP. Besides, variability in patient anatomy and disease progression makes it even more difficult to develop generalized and robust models for RP detection. A novel approach for dynamic intensity augmentation, conditional Generative Adversarial Network (cGAN), and self-supervised learning extracts meaningful features from limited datasets while simulating several pathological presentations.^5^ This is achieved by incorporating lung-specific adaptive augmentation and longitudinal data simulation; thus, this framework allows clinically plausible data to be generated, augmenting the training without introducing any artefacts that mislead the model. Moreover, radiomics-enhanced training embeds handcrafted features that include texture and shape descriptors with deep-learned features, which ensures better model interpretability and predictive performance.^6^ This study also utilized contrastive learning to further fine-tune feature representations and ensure that the model effectively used RP-specific patterns as opposed to healthy lung tissue.^7^ From the clinical point of view, radiation-induced pneumonitis diagnosis is highly reliant on human CT scan interpretation, which can be time-consuming and is subject to interobserver variability. RP during its early stages may be missed or misdiagnosed, especially in less resourceful environments.

## Methods

This section presents a framework specifically designed to address the challenge of limited annotated datasets for RP detection. The proposed approach enhances both detection and prediction capabilities by integrating advanced data augmentation, synthetic data generation, and self-supervised learning techniques.

### Data preparation

In the present work, one major and diverse CT-DICOM image dataset was considered, containing both verified lung cancer cases and normal controls. The database comprised 2963 axial CT slices from NSCLC patients and 383 slices from normal patients from publicly available datasets, ie, the NSCLC-Radiomics database (422 NSCLC patients), RIDER datasets (32 NSCLC patients), and clinical private repositories from a medical centre that collaborated with our laboratory (30 healthy controls), which included a diverse range of CT scans and radiographs of patients undergoing chest radiotherapy for various thoracic malignancies.^8^^,^^9^ The slices were derived from 3D CT volumes acquired on routine, non-contrast-enhanced chest CT scans. No 4D or dynamic scans were used, and all scans were reconstructed in the axial plane with uniform acquisition parameters between sites. The imaging data in CT used in this work included images of both RP-affected and healthy lungs. Since the number of RP-affected annotated datasets is extremely limited, publicly available CT images were supported with synthetic data created with new augmentation techniques and cGANs. Lung segmentation masks were generated using a pre-trained U-Net to allow subsequent analysis and augmentation to focus exclusively on the lung regions.^10–15^

#### Advanced data augmentation techniques

**Figure 1.**
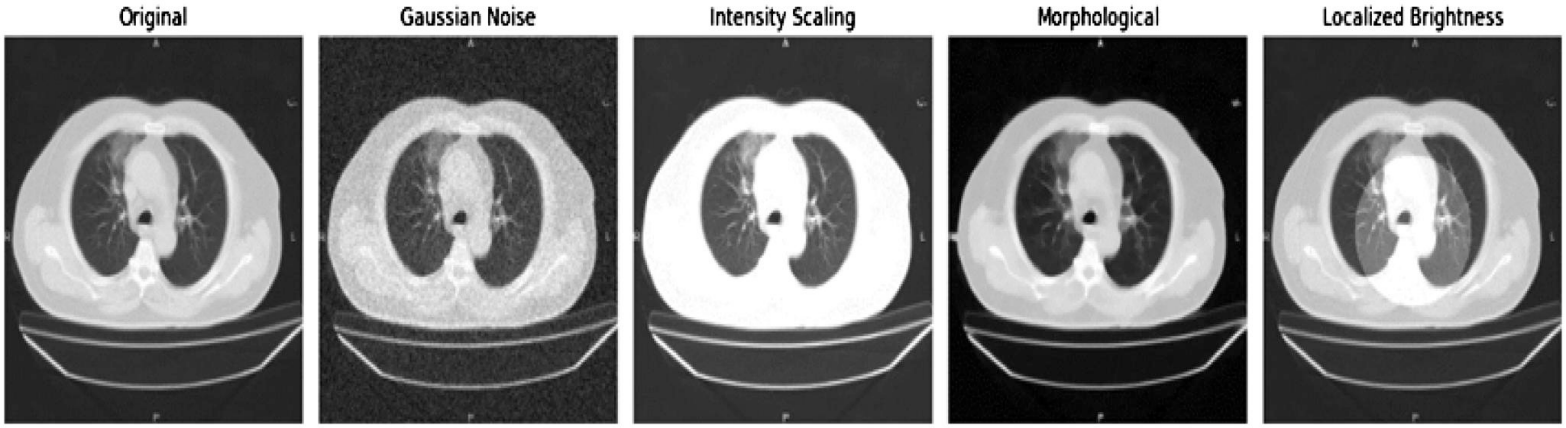
Augmented images with realistic lung tissue variations.

In order to increase the diversity of the training dataset, some augmentation techniques were utilized:

Dynamic intensity augmentation: this strategy simulated the changes in lung density caused by RP and added Gaussian noise specific to the region and morphological transformations to simulate both fibrosis and oedema.^16^  Figure 1 presents augmented images with more realistic variation in lung tissue densities, increasing the exposure of the model to a greater variety of pathological patterns.

Masked lung-specific augmentation: lung masks allowed for the application of transformations targeted, such as rotation, elastic deformation, and noise addition, restricted to clinically relevant lung regions, while preserving a high degree of anatomical plausibility.^17^ Longitudinal data simulation: successive scanning synthesizes the temporal development of RP, introducing smooth intensity variations that model early to late-stage RP.^18^ Augmentation techniques and the influence thereof on dataset diversity are summarized in Table 1.

**Table 1.** Augmentation techniques and their impact on dataset diversity.

Augmentation technique	Description	Impact on dataset diversity
Gaussian noise^20^	Introduces random pixel intensity variations to simulate density changes.	Increases the variability in tissue appearance, mimicking RP-related noise.
Intensity scaling^21^	Adjusts global brightness and contrast.	Simulates varying imaging conditions and tissue density changes.
Morphological transformations^22^	Applies operations (eg, dilation, closing) to alter texture.	Mimics structural changes such as fibrosis and oedema.
Localized brightness variation	Enhances or reduces intensity in specific regions.	Simulates focal pathological changes in lung tissue.
Elastic deformation^23^	Applies spatial warping to distort lung regions.	Captures anatomical variability and simulates physical deformation.
Rotation and flipping	Rotates or mirrors the lung images.	Ensures invariance to orientation and captures different viewing angles.

Abbreviation: RP = radiation-induced pneumonitis.

#### Synthetic data generation using conditional GANs

Finally, a cGAN was used to generate synthetic RP images conditioned on the provided lung segmentation masks.^19^

**Figure 2.**
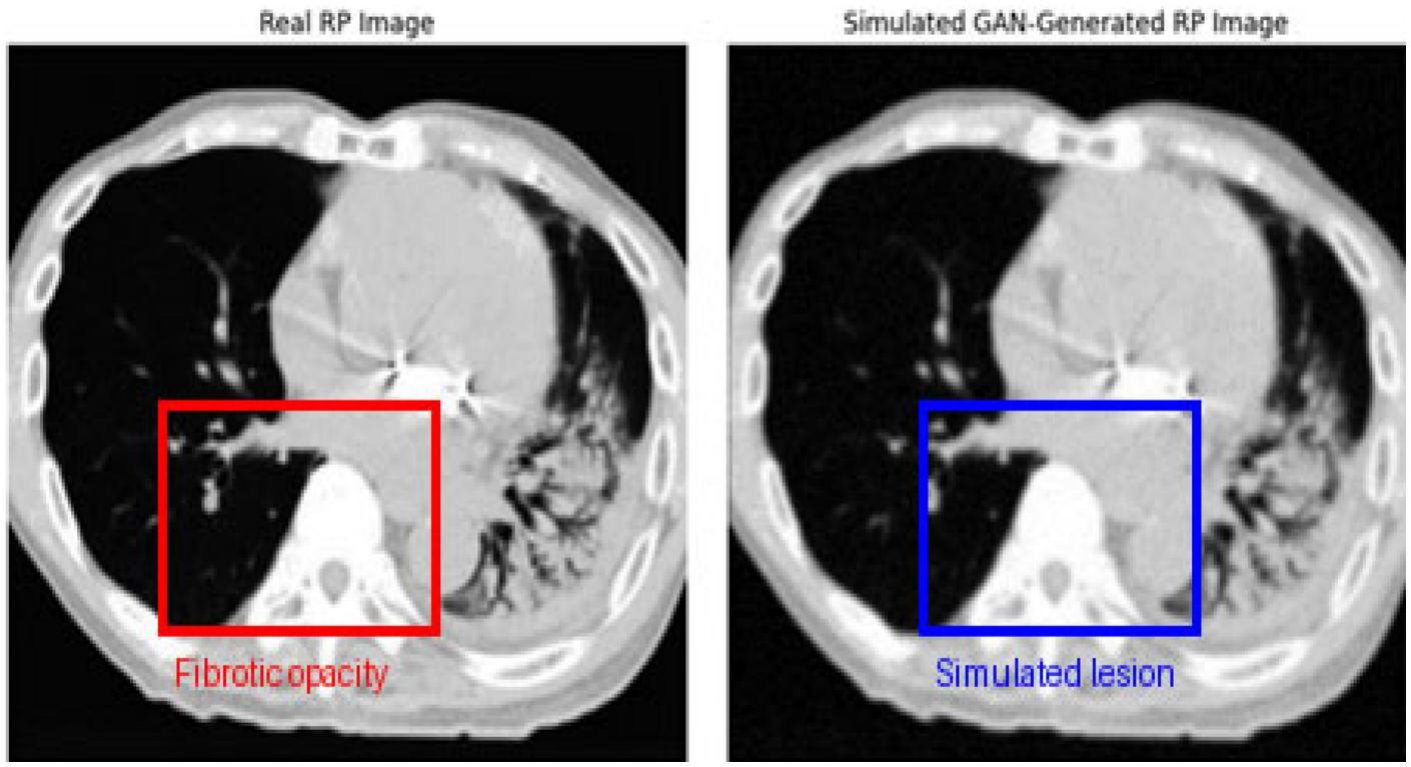
Real and GAN-generated RP images. Images achieved a Structural Similarity Index of 0.912, mean absolute error of 8.35, peak signal-to-noise ratio of 29.7 dB, and normalized cross correlation of 0.89. Abbreviation: GAN = Generative Adversarial Network; RP = radiation-induced pneumonitis.

The generator created a realistic variability of the RP pattern, taking as input pairs of healthy and RP-affected images. At the same time, this output was fed into the discriminator to assess the validity of the generated image. For both real RP and GAN-generated synthetic RP, some examples are shown in Figure 2; these are visually very consistent. A visual and quantitative comparison confirms the similarity between the 2: the synthetic images achieved a Structural Similarity Index of 0.912, mean absolute error of 8.35, peak signal-to-noise ratio of 29.7 dB, and normalized cross correlation of 0.89, indicating strong structural and intensity correspondence. These strongly results support the use of GAN-generated images as a valid augmentation strategy in RP detection pipelines.

### Self-supervised learning framework

**Figure 3.**
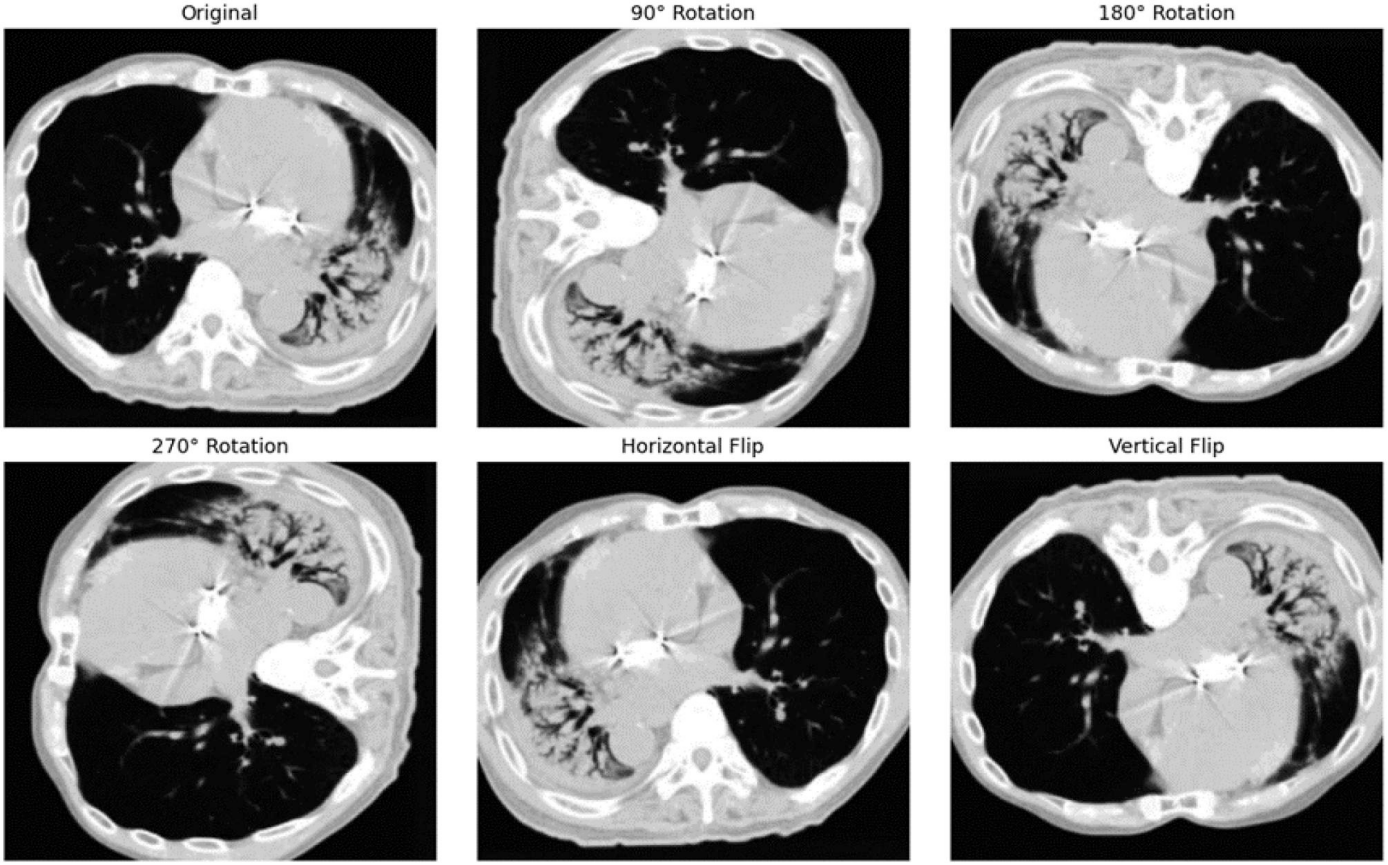
Self-supervised pretext tasks showing rotation prediction and flipping identification.

To fully make use of the unlabelled data, we used self-supervised learning for feature extraction.^24^ Self-supervised learning (SSL) allowed the model to learn the structural and textural features of lung anatomy associated with RP with no explicit labelling, as illustrated in Figure 3.

#### Pretext tasks

We proposed pretext tasks such as rotation prediction and the identification of image flipping, which support the model training on the spatial and structural patterns of the CT images.

#### Fine-tuning for RP detection

On the other hand, post-SSL pre-training, the model was fine-tuned on the labelled RP dataset, where it benefited from the learned representations focused on lung structures and patterns.

### Radiomics-augmented deep learning

Texture, shape, and intensity distributions were extracted using PyRadiomics from radiomic features combined with deep learning embeddings to improve interpretability and predictive performance.^11^  Equation 1 illustrates the inclusion of radiomics features in the deep learning pipeline as follows:


(1) $$F_{\mathrm{final}}=\alpha *F_{\mathrm{radiomics}}+\beta *F_{\mathrm{deep}},$$

where ***F*_final_** is the final feature representation, ***F*_radiomics_** and ***F*_deep_** are the radiomic and deep learning features, respectively, ***α*** and ***β*** are weighting factors learned during training.

#### Contrastive learning for feature representation

To refine RP-specific feature representations, contrastive learning was employed. It was trained on positive pairs of augmented RP images—and negative pairs of healthy RP images—to learn how to tell apart RP patterns from normal lung textures. The loss function for contrastive learning is defined as:


(2) $$L_{\mathrm{contrastive}}=\frac{1}{N}\sum_{i=1}^{N}\left[y_{i}*d_{i}^{2}+(1-y_{i})*\max{\left(0,m-d_{i}\right)}^{2}\right],$$

where

- ***y_i_*** indicates whether the pair is positive (1) or negative (0),
- ***d_i_*** is the distance between feature embeddings, and
- **m** is the margin parameter.

#### Spatial attention mechanisms

**Figure 4.**
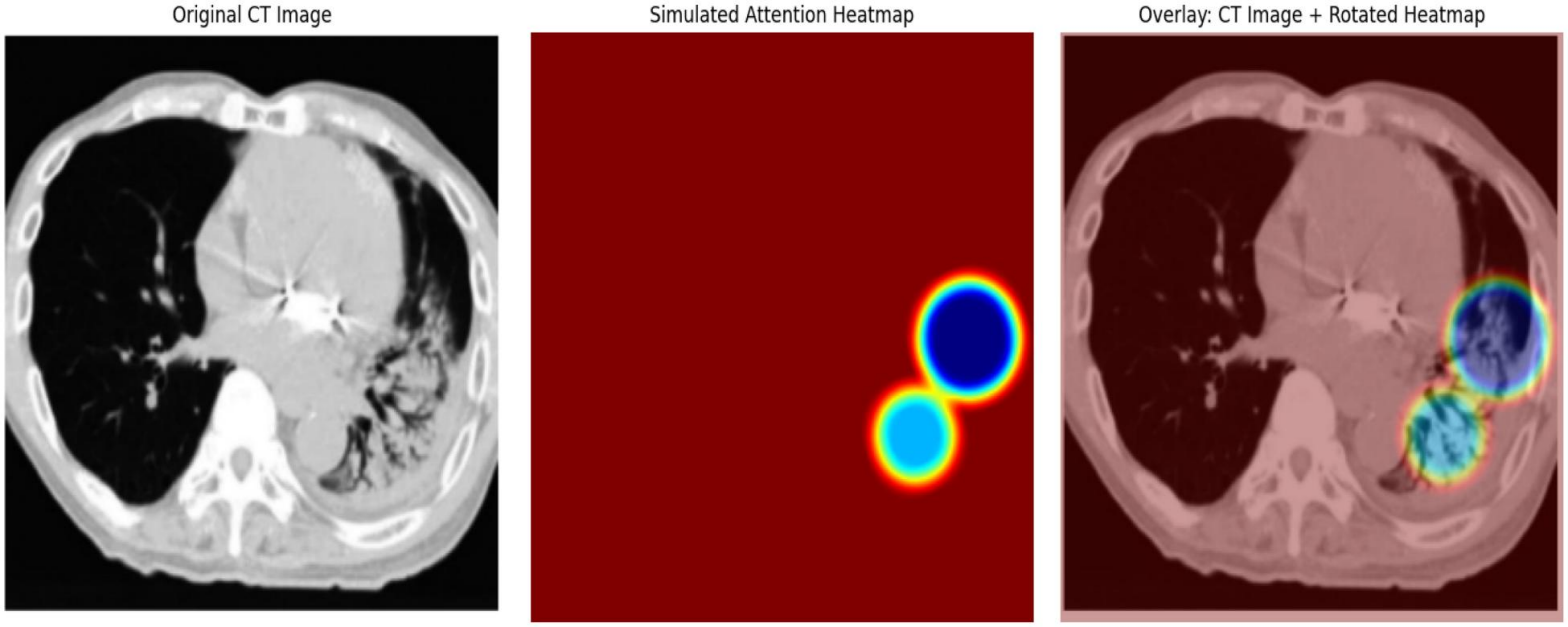
Spatial attention mechanisms with Grad-CAM.

Spatial attention mechanisms were incorporated to enhance part of the localization accuracy by focusing on regions most likely affected by RP.^25^ Attention maps, visualized in Figure 4, provide very clear indications of where high-risk areas are, hence aiding model interpretability and providing further insight to radiologists.

### Model training and cross-validation

The model was trained on the resulting augmented dataset containing real, synthetic, and augmented images. Some of the loss functions used in segmentation are Dice coefficient loss, whereas for classification, it is usually binary cross-entropy.

For model robustness evaluation, a 5-fold cross-validation strategy was employed, with performance metrics such as accuracy, precision, recall, F1-score, and area under the curve, among others, as shown in Table 2.

**Table 2.** Model performance metrics across 5-fold cross-validation.

Metric	Fold 1	Fold 2	Fold 3	Fold 4	Fold 5	Mean ± SD
**Classification**						
Accuracy (%)	91.4	92.1	93.0	90.8	91.7	91.8 ± 0.81
Precision (%)	89.5	91.0	92.2	89.0	90.2	90.4 ± 1.25
Recall (%)	90.8	92.5	93.8	91.2	91.8	92.0 ± 1.07
F1-score (%)	90.1	91.7	93.0	90.0	90.9	91.1 ± 1.16
AUC	0.94	0.95	0.96	0.93	0.94	0.94 ± 0.01
**Segmentation**						
Dice similarity coefficient (DSC)	0.86	0.87	0.88	0.85	0.87	0.87 ± 0.01
Jaccard Index	0.78	0.79	0.80	0.77	0.79	0.79 ± 0.01
Precision (%)	88.1	89.3	90.2	87.5	88.8	88.8 ± 1.04
Recall (%)	85.0	85.9	87.4	84.6	85.7	85.7 ± 0.96
F1-score (%)	86.4	87.5	88.8	85.8	87.2	87.1 ± 1.03

Abbreviation: AUC = area under the curve.

The performance of the proposed model was evaluated comprehensively by carrying out 2 tasks: classification and segmentation.

#### Uncertainty quantification for clinical reliability

The Bayesian layers were added to quantify the uncertainty of the predictions, with Monte Carlo dropout during inference providing confidence intervals for model predictions; hence, the model could express calibrated uncertainty and maintain reliable performance in ambiguous cases. This is particularly important in clinical settings, where low-confidence predictions should be subjected to further review.^26^

## Results

This paper demonstrated how newer methodologies can successfully overcome data limitations in training machine learning models reactive to RP diagnosis and prediction.

### Enhanced data generation and model performance

Integration of GAN-augmented and GAN-generated images with real CT scans augmented the diversity of the training dataset to enable better generalization of the model across fine RP presentations. The makeup of the training dataset-real samples, augmented samples, and synthetic samples-is presented in Table 3, showing their relative ratios.

**Table 3.** Dataset composition and model performance.

Dataset type	Images (%)	Model accuracy
Real	50%	88.5%
Augmented	35%	91.2%
GAN-generated	15%	92.6%
Combined (all sources)	100%	94.8%

Abbreviation: GAN = Generative Adversarial Network.

Real data, which forms 50% of the dataset, serves as a strong baseline with 88.5% accuracy, but further inclusion of augmented and GAN-generated data at 35% and 15%, respectively, boosted that performance, and the combined dataset achieved 94.8% accuracy. These results point to the importance of balanced dataset composition, with the synergy of real, augmented, and synthetic data for improved model robustness and generalization, making their way into scalability applications in medical imaging.

### Interpretability through multi-level attention mechanisms

**Figure 5.**
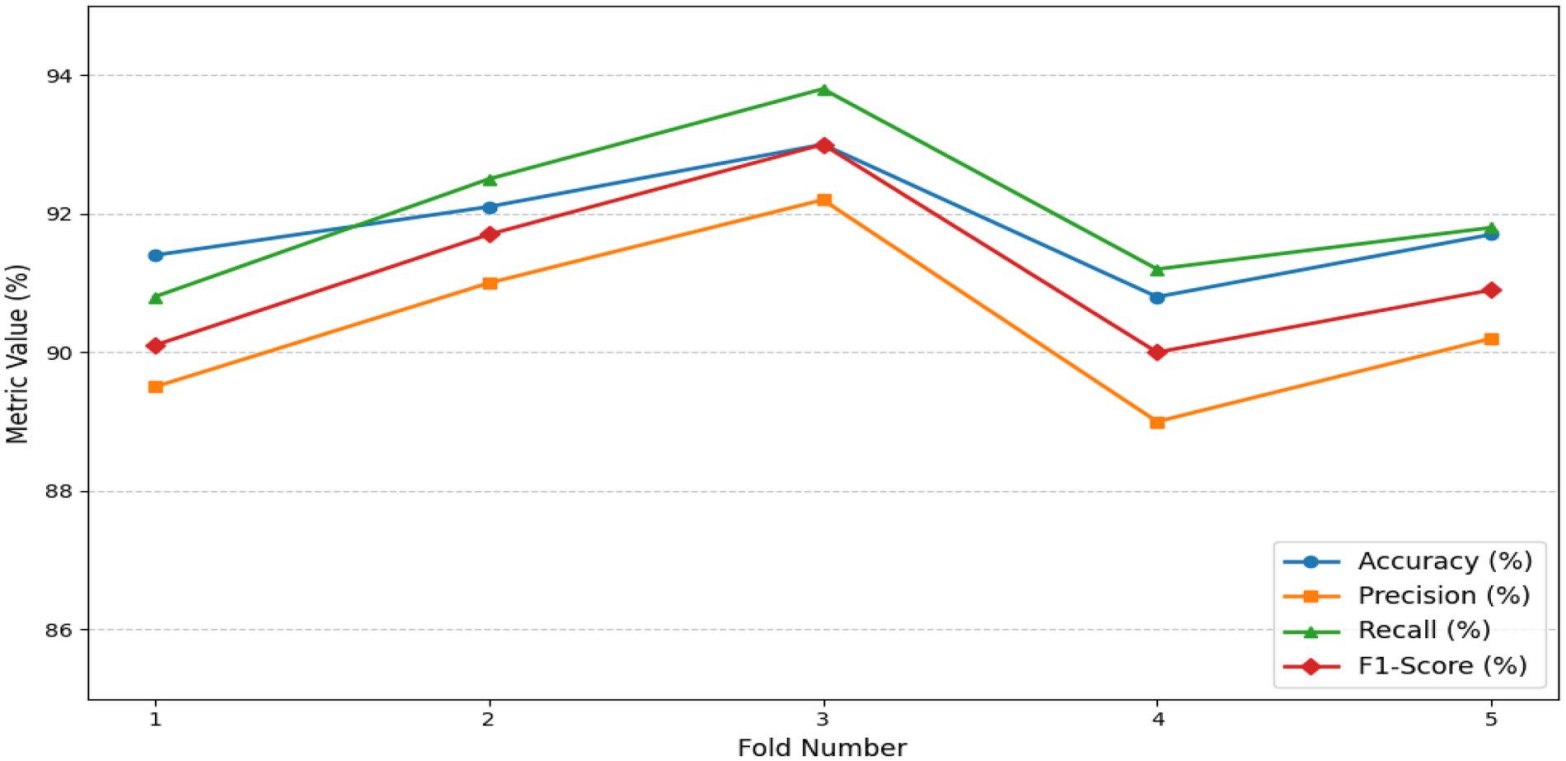
Performance trends across 5-fold cross-validation.

While including the spatial and channel attention mechanisms, this allowed the model to pay attention to the most informative region within the CT images. Table 4 provides quantitative results on the alignment between the predicted attention regions and the ground truth annotations. Figure 5 depicts the performance trend in terms of accuracy, precision, recall, and F1-score regarding the robustness and stability of the 5-fold cross-validation of the proposed model.

**Table 4.** Localization metrics using attention mechanisms.

Metric	Value
Intersection over union (IoU)	0.83
Dice coefficient	0.89
Average precision (AP)	0.91
Recall	0.87

### Robustness analysis across data variations

The stratified cross-validation was done across diverse datasets in order to test the model and assess its robustness. In doing this, it ensures the model does comparably well across the different splits of data and across individual demographic splits. It performed very well, with very small variability in accuracy and other key metrics. It highlighted that the approach generalizes well.

None of these varied metrics vary much between the folds: accuracy ranges between 90.8% and 93.0%, while the F1-score varies between 90.0% and 93.0%, which suggests that the models are highly stable. This stability points to the effectiveness of the composition of the dataset, integrating real images with augmented images and those produced by GAN, as confirmation of the generalization capability of the model.

Table 5 shows the strong consistency of the model in terms of the 5-fold validation strategy. The accuracy of the classification task was 91.7%, and the F1-score for it was above 89.9%, showing very strong precision and recall balance. The achieved Dice coefficient in the segmentation by the model was as high as 87.6% with an intersection over union of 79.8%, which attests to the accurate localization of the RP-affected regions.

**Table 5.** Cross-validation metrics for classification and segmentation tasks.

Task	Metric	Fold 1	Fold 2	Fold 3	Fold 4	Fold 5	Mean ± SD
Classification	Accuracy (%)	91.4	92.1	91.7	90.8	92.5	91.7 ± 0.65
F1-score (%)	89.6	90.3	90.0	89.2	90.8	89.9 ± 0.60	
Segmentation	Dice (%)	87.2	88.1	87.5	86.8	88.3	87.6 ± 0.58
IoU (%)	79.4	80.1	79.7	79.2	80.5	79.8 ± 0.47	

Abbreviation: IoU = intersection over union.

### Key observations from radiomics integration

Incorporation of radiomics features into a deep learning pipeline augmented performance in both classification and segmentation tasks. For example, texture-based features enhanced the detection of RP in regions where subtle density changes occurred. Features having shape helped the model in differentiating RP-affected and non-affected regions.

Table 6 shows the impact of integrating radiomics features into the deep learning pipeline on the overall model performance. Adding handcrafted radiomics features to the deep embeddings significantly enhanced the classifier metric: accuracy increased from 89.2% (deep features only) to 93.4%, with the F1-score similarly improving from 88.7% to 93.0%. The radiomics features, as represented by the texture and shape, captured subtle variations in the regions affected with RP and appeared complementary to the abstract representations learned by a deep network. This synergistic combination enhanced the model’s ability to discriminate between RP and non-RP events, especially in complex situations. The integration of real, augmented, and GAN-generated datasets, including radiomics features, and using the attention mechanism resulted in a model that offered high accuracy along with interpretability.

**Table 6.** Impact of radiomics on model metrics.

Feature type	Accuracy (%)	Precision (%)	F1-score (%)
Deep features only	89.2	88.3	88.7
Radiomics only	86.7	85.5	86.0
Combined features	93.4	92.5	93.0

## Discussion

This work investigates a novel approach to overcoming the basic data limitation problems inherent in diagnosing and prognosticating RP, a critical side effect of radiation therapy in lung cancer patients. Integration of GAN-based data generation, SSL, the incorporation of radiomics, and spatial attention mechanisms can provide great opportunities for leveraging novel techniques that enhance the robustness of the model, its accuracy, and interpretability. Other than classical augmentation, GANs provided new and realistic variations that enriched the dataset while maintaining consistency in visual and diagnostic appearance. While this mainly overcame the challenges posed by limited annotated data, it also proved instrumental for enhancing the generalization capability of the model across unseen samples. Fully, the overall gain in accuracy and robustness through this type of model justifies the efficiency of synthetic data use within training workflows, particularly for rare or underrepresented conditions such as RP. Spatial and channel attention mechanisms greatly enhanced model interpretability by underlining high-risk regions within CT scans. Attention maps showed a great correlation with the radiologists’ annotations, thus allowing an improvement in localizing areas affected by RP. This enhanced model interpretability is considered essential for eventual clinical adoption, wherein healthcare professionals need to understand and trust AI-driven diagnostic tools. The cross-validation results of 5-fold emphasized the robustness of the proposed methodologies, where the performance in most folds showed very little variation. The inclusion of synthetic data was noteworthy, showcasing the effectiveness of both augmented and GAN-generated datasets in improving generalization without introducing significant bias.

## Conclusion

Integration of GAN-generated images, SSL, and radiomics inclusion propelled the proposed methodologies to achieve much-enhanced improvements in model performance, interpretability, and robustness.
